# Silver Nanoparticles Decorated with PEGylated Porphyrins as Potential Theranostic and Sensing Agents

**DOI:** 10.3390/ma14112764

**Published:** 2021-05-23

**Authors:** Angelo Nicosia, Antonio Abbadessa, Fabiana Vento, Antonino Mazzaglia, Placido Giuseppe Mineo

**Affiliations:** 1 Department of Chemical Sciences and INSTM UdR of Catania, University of Catania, V.le A. Doria 6, 95125 Catania, Italy; angelo.nicosia@unict.it (A.N.); abbadessa.antonio@gmail.com (A.A.); fabiana.vento@phd.unict.it (F.V.); 2 CNR-ISMN, Istituto per lo Studio dei Materiali Nanostrutturati, V. le F. Stagno d’Alcontres 31, 98166 Messina, Italy; antonino.mazzaglia@cnr.it; 3 Institute for Chemical and Physical Processes CNR-IPCF, Viale F. Stagno d’Alcontres 37, 98158 Messina, Italy; 4 Institute of Polymers, Composites and Biomaterials CNR-IPCB, Via P. Gaifami 18, 95126 Catania, Italy

**Keywords:** AgNPs, PEGylated porphyrin derivative, covalent coating, acidity sensor, long-range alignment, reusable sensor

## Abstract

Silver nanoparticles (AgNPs) stand out over other metal nanoparticles thanks to their peculiar bactericidal and spectroscopic properties. Tunability of the AgNPs chemical–physical properties could be provided through their organic covalent coating. On the other hand, PEGylated porphyrin derivatives are versatile heteromacrocycles investigated for uses in the biomedical field as cytotoxic and tracking agents, but also as sensors. In this work, an easy multi-step approach was employed to produce coated silver nanoparticles. Specifically, the AgNPs were functionalized with 5,10,15-[*p*-(ω-methoxy-polyethyleneoxy)phenyl]-20-(*p*-hydroxyphenyl)-porphyrin (P(PEG350)_3_), using chloropropanethiol as a coupling agent. The P(PEG350)_3_ was structurally characterized through MALDI-TOF mass spectrometry, NMR spectroscopy and thermal analyses. The functionalization of AgNPs was monitored step-by-step employing UV-Vis spectroscopy, dynamic light scattering and thermogravimetric techniques. HRTEM and STEM measurements were used to investigate the morphology and the composition of the resulting nanostructured system (AgNP@P(PEG350)_3_), observing a long-range alignment of the outer porphyrin layer. The AgNP@P(PEG350)_3_ combines the features of the P(PEG350)_3_ with those of AgNPs, producing a potential multifunctional theranostic tool. The nanosystem revealed itself suitable as a removable pH sensor in aqueous solutions and potentially feasible for biological environment applications.

## 1. Introduction

Metal nanoparticles are among the nanotechnology keystones. Their peculiar properties make them useful in several scientific areas, such as nanomedicine, sensing, catalysis and optoelectronics [1]. Among them, silver nanoparticles (AgNPs) display multiple advantages: (i) higher extinction coefficient [2] than other noble metal nanoparticles having similar size, (ii) broad-spectrum bactericidal properties [3,4] and (iii) the possibility to be coupled with other nanosystems to obtain advanced materials [5]. In this direction, researchers have faced the issue of surface functionalization, employing several pathways and chemicals [2,6].

The AgNPs-based systems have been investigated in biological sensing [7,8] and imaging [9,10], exploiting their tunable optical properties lying on the localized surface plasmon resonance (LSPR). On the other hand, despite that the cytotoxicity of AgNPs leads to cellular apoptosis [10], the biocompatibility of these nanosystems could be tuned by suitable coatings, leading to their applications in photothermal therapy [11] and theranostics [12,13].

Porphines are natural pyrrolic-based heteromacrocycles with antenna properties for solar light radiation and thus have a fundamental role in nature and biological processes, such as photosynthesis. Synthetic porphyrin derivatives have been developed to exploit the unique features of these systems. They have been efficiently applied as reactive singlet oxygen (ROS)-generating agents in photodynamic treatments [14], but their usefulness has also been revealed in non-linear optics [15] and molecular sensing. Interaction of the porphyrin unit with DNA paved the way to monitoring DNA local structure changes through porphyrin-based optical probes [16].

Nevertheless, ionic groups would affect the sensing properties and the chemical–physical interactions towards biological substrates [17,18,19]. Besides, the ionic groups are not exploitable for the covalent linkage of the porphyrin onto a substrate.

In this landscape, water-soluble PEGylated porphyrin derivatives represent a suitable alternative that has demonstrated its usefulness in different applications [20,21,22,23,24,25,26]. In particular, the PEG chains covalently linked to the porphyrin core ensure solubility in both biological environments and the most common organic solvents. Moreover, the PEGylation of the heteromacrocycle provides stealth properties to the porphyrin core [27,28,29], which could increase body circulation time in therapeutic administration. Indeed, these porphyrin derivatives have been investigated for photodynamic therapy (PDT) applications, thanks to their ability to produce reactive singlet oxygen species under suitable light irradiation [30]. Recently, the coupling of two different porphyrins into a novel PEGylated penta-core derivative resulted in a nanobomb suitable as a surgical tool [31].

Moreover, PEGylated porphyrin derivatives have shown their biological sensing efficacy [17,24,32] and xenobiotic molecules’ sensing [33].

In the sensing field, PEGylated porphyrins revealed themselves useful to monitor pH variations in organic environments through spectroscopic variations [34]. Such feature has also been exploited in solid-state through the covalent link of the porphyrin moiety onto suitable substrates [35,36,37].

In this work, a three-step covalent coating of AgNPs with 5,10,15-[*p*-(ω-methoxy-polyethyleneoxy)phenyl]-20-(*p*-hydroxyphenyl)-porphyrin (P(PEG350)_3_) through 3-chloro-1-propanethiol is proposed. The goal is to couple the AgNPs’ bactericidal and photothermal properties with the features of the PEGylated porphyrins. In this hybrid system, PEG chains enhance the stability in solution of the metal nanoparticles, besides providing stealth ability [38]. Indeed, the porphyrin moiety could be exploited as a ROS generating agent and, thanks to its peculiar spectroscopic features (high molar absorption and strong fluorescence), it could also be used as a tracking agent in biological environments.

A complete characterization of the nanosystem and of the precursors was performed, employing a plethora of techniques (MALDI-TOF, NMR, UV-Vis, fluorescence, DLS, DSC, TGA, TEM and STEM). Finally, the sensing ability to acidity variation was ascertained by spectroscopic measurements.

## 2. Materials and Methods

Poly(ethyleneglycol)methyl ether, thionyl chloride, silver nitrate, sodium borohydride, 3-chloro-1-propanethiol, PBS solution, 5,10,15,20-tetrakis(4-sulfonatophenyl)porphyrin, *p*-nitroso-N,N’-dimethylaniline, hydrochloric acid (≥37%), tetrahydrofuran, chloroform, ethanol, triethylamine, water (LC-MS grade), acetone, acetone-d6 and *trans*-2-[3-(4-tert-Butylphenyl)-2-methyl-2-propenylidene] malononitrile were purchased from Sigma-Aldrich (Merck Group, Milan, Italy).

The 5,10,15,20-[*p*-(*ω*-methoxy-polyethyleneoxy)phenyl]-porphyrin (P(PEG750)_4_) was obtained as reported elsewhere [39].

### 2.1. Instrumentation

MALDI-TOF mass spectra were acquired with a Voyager DE (PerSeptive Biosystem, Perkin Elmer, Waltham, MA, USA), detecting the ions in linear mode by using the delay extraction device (25 kV applied after 2600 ns, a potential gradient of 454 V mm^−1^ and a wire voltage of 25 V) [40,41]. *Trans*-2-[3-(4-*tert*-Butylphenyl)-2-methyl-2-propenylidene] malononitrile (DCTB) was used as a matrix. Mass spectrometer calibration was performed as previously published [42]. Grams/386 software (Version 3.04, Galactic Industries Corp, Salem, NH, USA) was applied on the spectra to determine the weight-average molecular weights, according to a previously reported method [40].

^1^H-NMR spectra were acquired through a ^UNITY^INOVA instrument (Varian, Agilent Technologies, Santa Clara, CA, USA) operating at 500 MHz (^1^H), setting the sample temperature at 27 °C. VnmrJ software (Version 2.2C, Varian, Agilent Technologies, Santa Clara, CA, USA) was employed for spectra acquisition and processing. The sample was dissolved in acetone-d6 and the chemical shifts were expressed in ppm (compared to the acetone residue signal).

Thermogravimetric analyses were performed with a Perkin-Elmer TGA 7 equipped with a TAC 7/DX (Perkin Elmer, Waltham, MA, USA), in the range from 50 to 800 °C (thermal ramp of 10 °C min^−1^) and air atmosphere (60 mL min^−1^).

Differential scanning calorimetry (DSC) measurements were conducted with a TA Q20 instrument (TA instruments, New Castle, DE, USA), equipped with a Refrigerant Cooling System (RCS90, TA instruments, New Castle, DE, USA) (heating rate of 10 °C min^−1^, temperature range from −80 to 55 °C) and under an anhydrous nitrogen atmosphere (60 mL min^−1^).

UV-Vis spectra (Agilent Technologies, Santa Clara, CA, USA) were acquired with an Agilent Technologies Cary60 UV-Vis spectrophotometer, using quartz cuvettes (1 cm path length) and water or acetone as solvents (T = 25 ± 0.1 °C).

Fluorescence spectra were acquired with a FP-8200 spectrofluorimeter (Jasco Corporation, Tokyo, Japan), in quartz cells (1 cm path length) using water or acetone as a solvent (T = 25.0 ± 0.1 °C). A long-pass filter was placed along the emission path.

Dynamic light scattering (DLS) analyses were performed through a miniDAWN Treos multi-angle light scattering detector (Wyatt Technology, Santa Barbara, CA, USA), equipped with a Wyatt QELS DLS Module. The measurements were acquired using water or acetone solutions (T = 25 ± 0.1 °C) previously filtered with a 0.2 μm filter. Wyatt software (ASTRA 6.0.1.10) was used to calculate the size distributions.

The nanoparticles’ morphology was investigated with a transmission electron microscopy (TEM) JEM2100 LaB6 (200 kV) (JEOL, Peabody, MA, USA), and a digital scanning transmission electron microscopy, STEM (JEOL, Peabody, MA, USA), equipped with BF and DF STEM Detectors plus a SE/BSE detector. The samples were water-dispersed (~0.5 mg/mL) through an ultrasound bath (10 min); then, ten drops of water-suspension were placed on holey-carbon-coated copper grids (300 mesh). These measurements were performed at the University of Exeter, UK.

### 2.2. Synthesis of 5,10,15-[p-(ω-methoxy-polyethyleneoxy)phenyl]-20-(p-hydroxyphenyl)-porphyrin

The 5,10,15-[*p*-(ω-methoxy-polyethyleneoxy)phenyl]-20-(*p*-hydroxyphenyl)-porphyrin [P(PEG350)_3_] was synthetized according to our method [39], through a reaction between 5,10,15,20-tetrakis(*p*-hydroxyphenyl)porphyrin and chlorinated poly(ethylene glycol)methyl ether (PEGMEC) in the presence of NaOH. PEGMEC was prepared in tetrahydrofuran (THF) solution through a reaction between thionyl chloride and poly(ethyleneglycol)methyl ether (average molecular mass of 350 Da and a PDI of 1.02). The acidulated product of the reaction was dried under vacuum and placed in a vacuum-oven for 2 days at 80 °C. Finally, the obtained product was dissolved in chloroform (CHCl_3_) and fractionated by a chromatographic column, using silica gel as the stationary phase and a solution of CHCl_3_/C_2_H_5_OH/N(C_2_H_5_)_3_ (97.0:2.0:1.0) as the eluant. MALDI-TOF analysis (see Section 3) confirmed that P(PEG350)_3_ was the second compound eluted from the column with ~30% yield. ^1^H-NMR (Acetone-d6, ppm): a multiplet at 8.91 ppm (8 H, C-H pyrrole protons in 2,3,7,8,12,13,17,18), two doublets at 8.13 ppm (6 H, C-H phenyl protons, *a*) and at 8.08 ppm (6 H, C-H phenyl protons, *a′*), two doublets at 7.37 ppm (2 H, C-H phenyl protons, *b*) and 7.32 ppm (2 H, C-H phenyl protons, *b′*) and a singlet at −2.66 ppm (2 H, N-H pyrrole protons). The signals due to the poly(ethylene glycol) (PEG) arms were: an unresolved multiplet between 4.46 and 3.31 ppm (about 108 H, -CH_2_- groups of the PEG branches, *c + d+PEO*) and a broad peak at 3.26 ppm (9 H, the -OCH_3_ terminal groups of the branches). MALDI-TOF: Mw = 1743, Mn = 1725, PDI = 1.01.

### 2.3. Synthesis of Silver Nanoparticles

Silver nanoparticles (AgNPs) were synthesized in an aqueous solution through chemical reduction of silver nitrate [43]. Briefly, a sodium borohydride water solution (1.5 L, 2 mM) was prepared; subsequently, a silver nitrate water solution (0.5 L, 1 mM) was added under high-speed stirring through a dropping funnel. To assure the reduction of the whole silver salt added and to stabilize the nanoparticles’ suspension, the mixture was stirred for 30 min. The so-obtained AgNPs colloidal suspension was stored in the fridge (5 °C). UV-Vis measurements have checked the localized surface plasmon resonance (LSPR) signal of such AgNPs at 394 nm.

### 2.4. Functionalization of the Silver Nanoparticles with 3-Chloro-1-Propanethiol

Briefly, HCl 1M was added to an aqueous solution (10 mL) of AgNPs (0.27 mg), regulating the pH at ~3.7. Subsequently, 40 µL (4.1 × 10^−4^ mol) of 3-chloro-1-propanethiol were added. The mixture, heated at 60 °C, was left under strong stirring for 3 h.

Then, the solution was cooled to room temperature and centrifuged (5000 rpm, 15 min). The obtained residue was sonicated in ethanol and centrifuged (2 times repeated). Finally, the product AgNP@ClPT was dried under vacuum (60 °C, 24 h).

### 2.5. Covalent Monolayer Coating of AgNP@ClPT with P(PEG350)_3_

Briefly, AgNP@ClPT (5 mg) were dispersed in 10 mL of acetone also containing 5 μL of triethylamine and 5 mg of P(PEG350)_3_. The mixture was held at boiling point for 24 h. Then, to separate the unreacted P(PEG350)_3_, the solution was cooled at 25 °C and centrifuged (9000 rpm, 15 min) and the colored supernatant was eliminated. AgNP@P(PEG350)_3_ residue was suspended in acetone, sonicated for 5 min and centrifuged (2 times repeated). The final product AgNP@P(PEG350)_3_ was dried under vacuum (60 °C, 24 h).

### 2.6. Spectroscopic Titration

The spectroscopic titrations were performed employing HCl solutions (standardized with sodium carbonate used as a primary standard). To aqueous solutions (2.5 mL) of P(PEG750)_4_ and AgNP@P(PEG350)_3_, HCl solutions volumes from 0.2 up to 10 μL were added, aiming to reduce the dilution error. To monitor the titration in function of the H^+^ concentration, UV-Vis and fluorescence emission spectra were acquired after every addition of the acidic solution. The dilution effect was considered by correcting the spectra after each addition.

### 2.7. Estimation of Singlet Oxygen Production

The ^1^O_2_ amount produced by the AgNP@P(PEG350)_3_ nanosystem was determined employing a standard method [44] involving the *p*-nitroso-N,N’dimethylaniline (RNO) bleaching reaction.

The photosensitizer AgNP@P(PEG350)_3_ was dissolved in PBS solution together with imidazole (10 mM) and RNO (50 mM). To attribute the time dependence of absorbance (λ = 440 nm) essentially to the RNO photo-chemical reaction, higher concentration of RNO than that of photosensitizer was employed. Then, the solution was irradiated with a green laser (λ = 532 nm, 50 mW).

To determine the quantum yield (Φ) of ^1^O_2_ formation for AgNP@P(PEG350)_3_, a solution of 5,10,15,20-tetrakis(4-sulfonatophenyl)-porphyrin (TPPS) was used as a standard reference and subjected to the same experimental conditions (Φ_TPPS_ = 0.62). The Φ was calculated using Equation (1):

Φ = Φ_TPPS_·(OD^TPPS^/OD^AgNP@P(PEG350)^_3_)·(ΔA/ΔA^TPPS^) (1)

where OD is the optical density at the laser irradiation wavelength for the photosensitizer solution and ΔA is the absorbance variation (440 nm) for AgNP@P(PEG350)_3_ solution, and OD^TPPS^ and ΔA^TPPS^ are the values for TPPS solution.

## 3. Results

Prior to the AgNPs functionalization, the 5,10,15-[*p*-(ω-methoxy-polyethyleneoxy)phenyl]-20-(*p*-hydroxyphenyl) porphyrin (P(PEG350)_3_) has been synthesized by etherification reaction between 5,10,15,20-tetrakis(*p*-hydroxyphenyl)porphyrin (P) and poly(ethylene glycol)methyl ether chloride (PEGMEC) [39].

The P(PEG350)_3_ compound was isolated through column chromatography and characterized with MALDI-TOF mass spectrometry, ^1^H-NMR spectroscopy and DSC.

**Figure 1:**
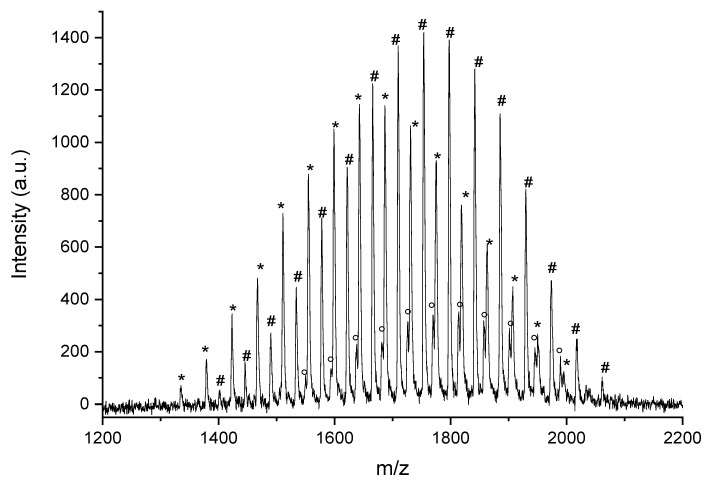
MALDI-TOF spectrum of P(PEG350)_3_, mtx DCTB. The symbols on the top of the peaks indicate the following species: #, [M]H^+^; *, [M]Na^+^; °, [M]K^+^.

The mass spectrum of P(PEG350)_3_ (Figure 1) shows two clusters of peaks, in particular at m/z values of 1337 + *n*44, 1403 + *n*44 and 1551 + *n*44, with *n* = 0–15, corresponding to the [M]H^+^ (#), [M]Na^+^ (*) and [M]K^+^ (°) species, respectively. A Mw value of 1743 Da (PDI = 1.01) was calculated from the data elaboration.

**Figure 2:**
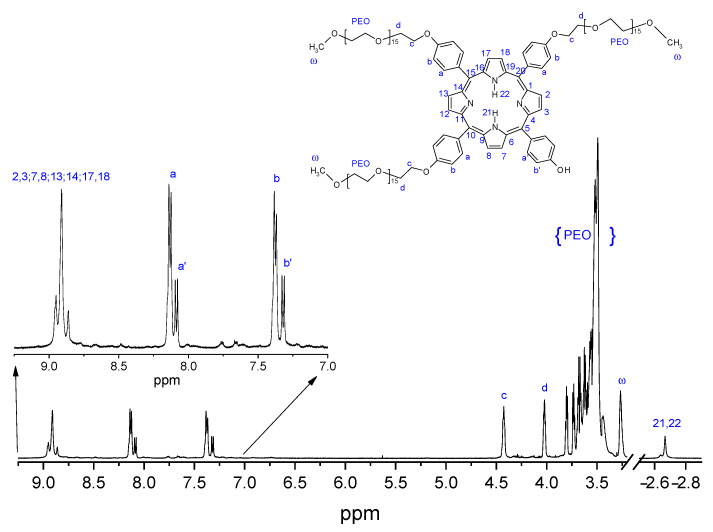
^1^H-NMR spectrum of P(PEG350)_3_, solvent Acetone-d6, 27 °C, 500 MHz.

NMR experiments also confirmed the chemical structure. In particular, the spectrum (Figure 2) shows the overlapped signals of the pyrrole protons at 8.91 ppm, arising from their different chemical surroundings. The signals at 8.08 and 7.32 ppm, attributed to protons *a′* and *b′*, further confirm the molecular structure. The presence of the PEG chains is revealed by the unresolved triplets between 4.46 and 3.4 ppm. Finally, at 3.25 ppm, the signal of methyl groups is also evident.

**Figure 3:**
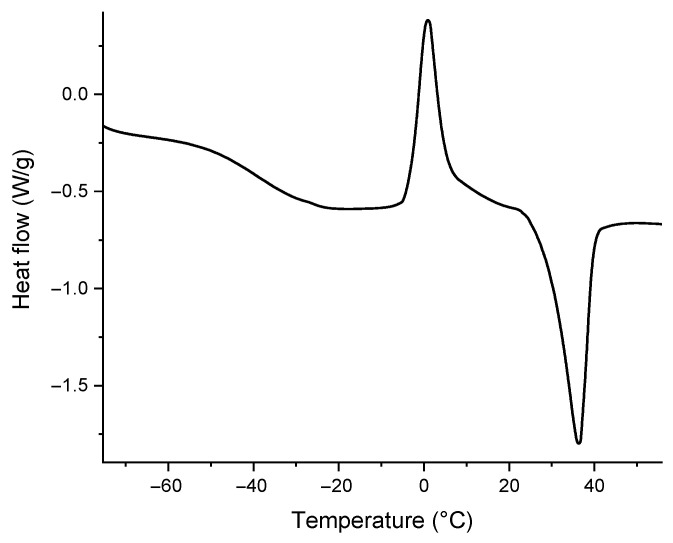
DSC trace of P(PEG350)_3_.

The DSC trace of P(PEG350)_3_, shown in Figure 3, revealed an unexpected thermal behavior with a glass transition temperature at −39.8 °C (Tg), followed by a devitrification phenomenon at 0.89 °C (ΔH = 10.0 J/g) and a subsequent melting event (Tm) at 36.3 °C (ΔH = 15.32 J/g), indicating the presence of both amorphous and crystalline phases. Taking into account the thermal properties of starting PEG350 (Tm below 0 °C) and P (Tm with decomposition at >300 °C), it is evident that the polymer functionalization effects the chemical–physical interactions involved in the thermal stability of the system. Indeed, due to the esterification of the P phenolic groups, hydrogen bonds between phenolic hydroxyl groups and pyrrolic nitrogen [45] lack in P(PEG350)_3_. Furthermore, the steric hindrance of the three PEG chains affects the π–π stacking interactions between porphyrin structures.

**Scheme 1:**
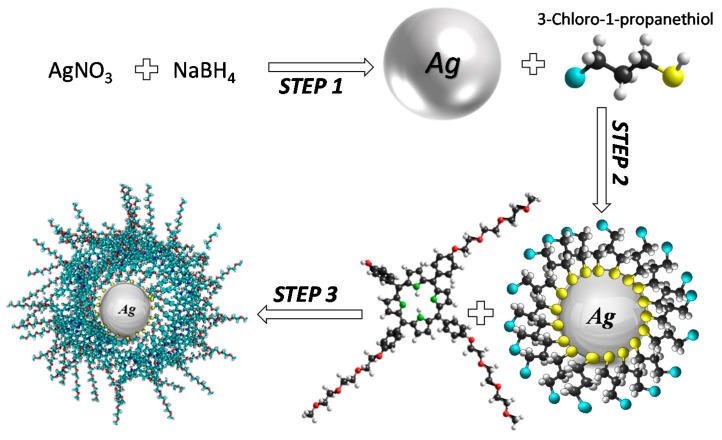
Simplified pathway for the silver nanoparticles’ functionalization.

The bottom-up approach to the synthesis of the silver nanoparticles (AgNPs) covalently functionalized with a PEGylated porphyrin derivative involves three steps: (1) AgNPs synthesis, (2) covalent coating with 3-chloro-1-propanethiol (AgNP@ClPT) and (3) covalent functionalization of the coated AgNPs with P(PEG350)_3_ (see Scheme 1).

**Figure 4:**
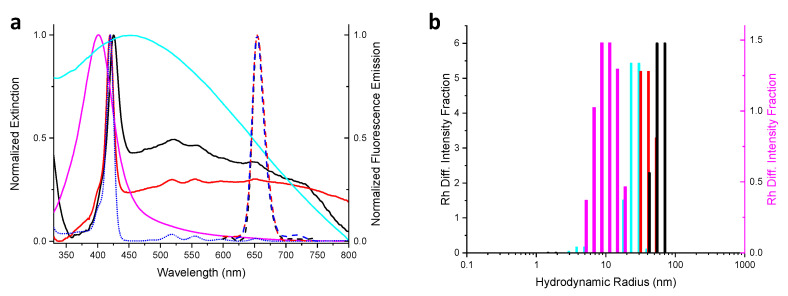
(**a**) UV-Vis spectrum of AgNPs (water, magenta line), P(PEG350)_3_ (acetone, dotted blue line), AgNP@ClPT (acetone, cyan line), AgNP@P(PEG350)_3_ in acetone (red line) and water (black line). Fluorescence emission spectra (λ_exc_ = 420 nm) of P(PEG350)_3_ (acetone, blue dashed line), AgNP@P(PEG350)_3_ in acetone (red dashed line) and water (black dashed line, overlapped with the blue one). (**b**) Hydrodynamic radius distribution of AgNPs (water, magenta), AgNP@ClPT (acetone, cyan), AgNP@P(PEG350)_3_ in acetone (red) and water (black).

The synthesis of AgNPs was performed through sodium borohydride-mediated reduction of silver nitrate (step 1) at room temperature. UV-Vis spectrum of AgNPs in water solution exhibited the typical localized surface plasmon resonance (LSPR) signal of AgNPs, centered at 401 nm (Figure 4a, black line), indicative of a diameter of ~15 nm [46]. The nanoparticles’ size was also confirmed by DLS measurements, revealing a mean hydrodynamic radius of 10.79 ± 3.07 nm (Figure 4b, magenta). Then, the obtained AgNPs were functionalized with 3-chloro-1-propanethiol (used as the coupling layer) in water solution at pH 3.7 (step 2). The AgNP@ClPT system was separated by centrifugation and further washed with ethanol. Once dried, the water-insoluble AgNP@ClPT sample was suspended in acetone and characterized through UV-Vis and DLS measurements. The UV-Vis spectrum (Figure 4a, cyan line) exhibited a broad band centered at 457 nm. The DLS analysis of functionalized nanoparticles revealed a hydrodynamic size of 25.9 ± 3.3 nm (Figure 4b, cyan). The broadening of the UV-Vis spectrum profile and the increased hydrodynamic size suggest a self-aggregation phenomenon of the AgNP@ClPT nanoparticles.

The porphyrin covalent coating of AgNP@ClPT (step 3) was performed in acetone in the presence of triethylamine. The reaction provides the nucleophilic substitution reaction between the hydroxyphenyl group of P(PEG350)_3_ and the chlorine of the AgNP@ClPT, as reported in Scheme 1. The AgNP@P(PEG350)_3_ nanoparticles were separated through centrifugation, further washed until obtaining a clear supernatant (checked by UV-Vis) and dried in a vacuum oven. The isolated hybrid system was suspended in both acetone or water and suitably characterized. The AgNP@P(PEG350)_3_ acetone solution exhibited the typical signals of the porphyrin moiety: a Soret band centered at 420 nm, due to the S_0_–S_2_ transitions, and four Q-bands in the region 500–675 nm, attributed to the S_0_–S_1_ transitions. It is evident that the porphyrin signals are overlapping with a broad signal attributed to the AgNPs LSPR band. The DLS measurement revealed a mean hydrodynamic radius of 40.0 ± 5.9 nm (Figure 4b, red), higher than that of AgNP@ClPT and confirming the covalent coating of the particles.

The AgNP@P(PEG350)_3_ resulted suspendable in water thanks to the hydrophilicity of the PEG chains of the porphyrin moieties. The related UV-Vis spectrum shows a Soret band centered at 427 nm, which is broader and red-shifted than that in acetone, due to the NPs aggregation and the solvatochromism effect of the porphyrin moiety [47]. DLS confirmed the NPs aggregation, revealing the increased hydrodynamic radius (56.6 ± 7.4 nm), compatible with inter-particles’ porphyrin aggregation (Figure 4b, black).

On the other hand, the fluorescence emission of the porphyrin moiety centered at 654 nm (λ_exc_ = 420 nm) seems not influenced by these phenomena (Figure 4a, red and black dashed lines).

**Figure 5:**
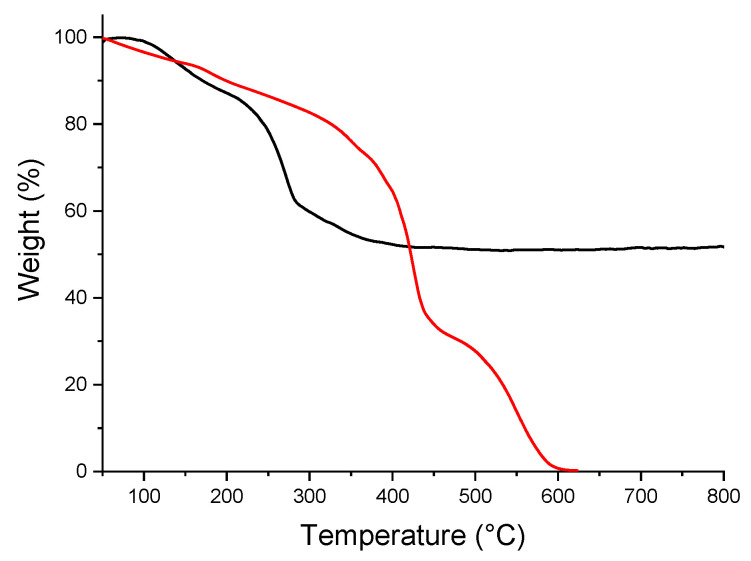
Thermogravimetric analysis (TGA) thermogram of AgNP@P(PEG350)_3_ (black line) and P(PEG350)_3_ (red line), in air atmosphere.

Thermogravimetric analysis of P(PEG350)_3_, conducted in air atmosphere, evidenced two degradative steps at about 390 and 518 °C, ending up at 618 °C, leaving any residue (Figure 5, red line). On the other hand, the AgNP@P(PEG350)_3_ shows the weight loss of the organic coating only, within the temperature range of 100–400 °C (Figure 5, black line). Since the AgNPs are thermostable, the residue (800 °C) of about 52% is due to the bare metal. Based on this data, the loading % of the organic layer was estimated to be about 48%. The downshift of the degradation temperatures with respect to the P(PEG350)_3_ suggests a possible two-step degradation involving the coupling layer moiety and/or a catalytic effect of the AgNPs towards the organic layer degradation.

**Figure 6:**
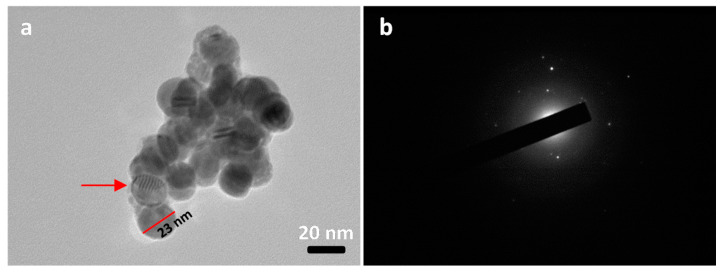
Representative transmission electron microscopy images of AgNP@P(PEG350)_3_ (**a**) deposited from water and the respective diffraction pattern (**b**).

**Figure 7:**
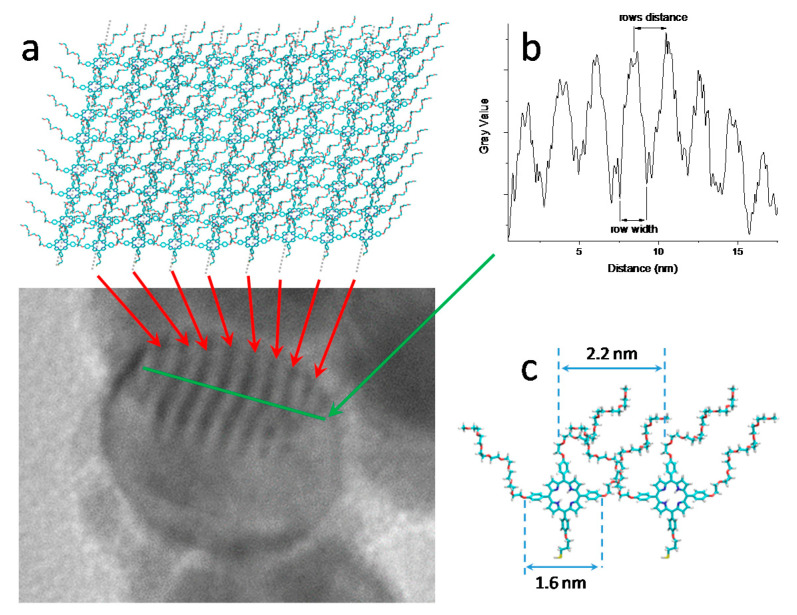
Simulated molecular nanopatterning of P(PEG350)_3_ bonded on AgNPs surface (**a**, top); magnification of AgNP@P(PEG350)_3_ TEM image (**a**, bottom); rows profile estimated in the green line path (**b**); MM^+^ molecular modeling simulations of nanostructured P(PEG350)_3_ (**c**).

The morphology and size of AgNP@P(PEG350)_3_ were studied through TEM experiments. The size of hybrid nanoparticles was revealed in the range of 15–30 nm (Figure 6a). The obtained average values also support the DLS results. Moreover, it is evident that the AgNPs structural properties were preserved in AgNP@P(PEG350)_3_, as confirmed by the diffraction pattern (Figure 6b). It is noteworthy to highlight the presence of nanopatterned structures onto the AgNP@P(PEG350)_3_ surface (Figure 6a, red arrow, and Figure 7a, bottom), evidenced by dark parallel rows overlapped to the functionalized AgNPs. The analysis of the nanopatterns (Figure 7b) revealed a profile having the mean row width of about 1.59 ± 0.17 nm and a distance between the rows’ centroid of about 2.14 ± 0.16 nm. Such evidence is attributable to the self-organization of the porphyrin molecules during the covalent linkage onto the metal nanoparticles. To confirm this hypothesis, a computer simulation of the edge-to-edge arrangement of two P(PEG350)_3_ molecules, obtained employing the Molecular Mechanics (MM+) algorithm, was performed. The calculation shows a width of the porphyrin structure (Figure 7c) of about 1.6 nm, and a distance between the adjacent porphyrins of about 2.2 nm (Figure 7c), confirming the hypothesized arrangement onto the metal nanoparticles. Moreover, a face-to-face self-organization determines the formation of the organized rows (Figure 7a, top). It is also noteworthy that the distance between the porphyrin planes in the face-to-face arrangement does not involve any transition dipole moment coupling (as confirmed by UV-Vis measurements, Figure 4a), probably because of the sterical hindrance of the PEG branches.

**Figure 8:**
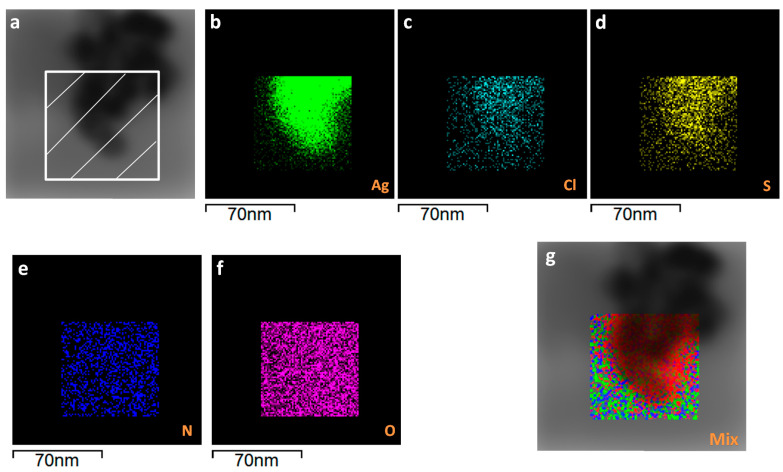
STEM of AgNP@P(PEG350)_3_ (**a**) with elemental analysis mapping (**b**–**f**) in dashed area of image (**a**) and their total merged composition (**g**).

Further confirmation of the AgNPs functionalization was pointed out through STEM measurements (Figure 8). The analysis exhibited the presence of sulfur, nitrogen and oxygen overlapped to the Ag signals, confirming the functionalization of the metal nanoparticles with the P(PEG350)_3_. Moreover, as expected, the residual chlorine signal suggests that the AgNPs porphyrin coating was not fully completed because of the steric hindrance of the PEGylated porphyrin derivative and the related “foot-print” (intended as the surface occupied by the P(PEG350)_3_).

**Figure 9:**
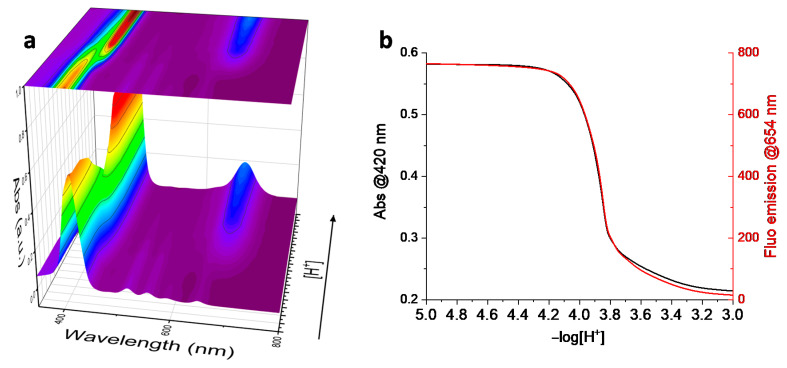
UV-Vis HCl-diluted solution titration of P(PEG750)_4_ water solution (5 μM): (**a**) 3D representation of the UV-Vis spectra acquired during the H^+^ titration; (**b**) titration curve obtained monitoring the UV-Vis absorbance at 420 mn (black line) and the fluorescence emission intensity at 654 nm (red line, λ_exc_ = 420 nm).

To study the sensing capabilities of the AgNP@P(PEG350)_3_ towards pH variations, titrations with dilute HCl solutions were conducted on both the water solutions of P(PEG750)_4_ (used as reference) and AgNP@P(PEG350)_3_. Since the pyrrolic nitrogen of the porphyrin core is subjected to protonation in acidic conditions, huge spectroscopic variations due to the molecular orbital modification level were expected. As an example, the spectroscopic titrations of P(PEG750)_4_ (used as a model) in water solution (5 μM) showed the disappearance of the band centered at 420 nm, together with the related four Q-bands, and the appearance of two new bands at 447 and 680 nm (Figure 9a, reporting the UV-Vis spectra variations as a function of H^+^ concentration). The 420 nm intensity band variation analysis revealed a flex point at pH of 3.86 (Figure 9b, black line), similar to the literature results [48].

The porphyrin core protonation also resulted in a strong decreasing of the fluorescence emission intensity at 654 nm (Figure 9b, red line). Spectrofluorimetric titrations represent a more accurate signal than the UV-Vis to monitor the pH variations due to the strong fluorescence emission. The occurrence of the titration flex point at pH 3.86 was further confirmed.

**Figure 10:**
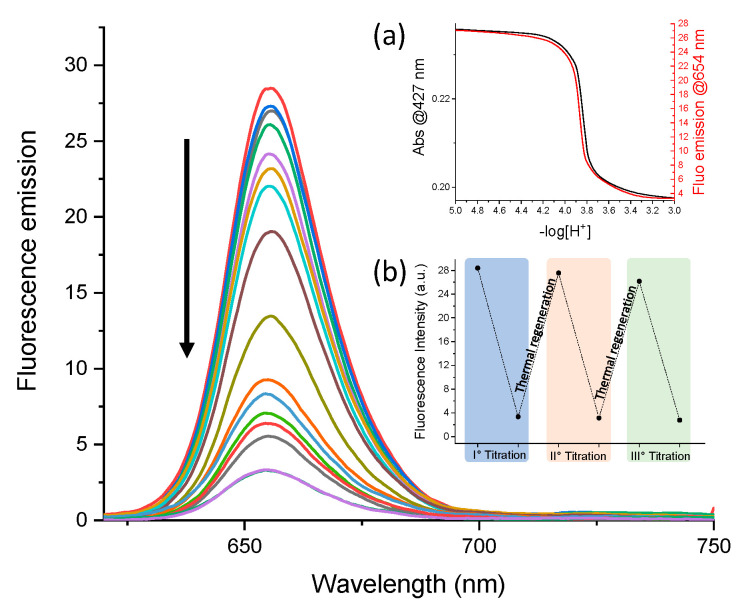
Spectrofluorimetric HCl titration of AgNP@P(PEG350)_3_. Inset: (**a**) titration curves obtained monitoring the fluorescence emission intensity at 654 nm (red line) and the UV-Vis absorbance at 427 nm (black line); (**b**) fluorescence emission intensity variations (at 654 nm) of AgNP@P(PEG350)_3_ aqueous solution during HCl titrations cycles.

The linkage of the porphyrin derivative moiety to the AgNPs does not alter the porphyrin spectroscopies properties and the pK of the system. As revealed from the titrations performed on AgNP@P(PEG350)_3_, the flex point at the same acid concentration value was recorded employing both fluorescence emission at 654 nm (λ_exc_ = 420 nm) and UV-Vis (signal at 427 nm) spectroscopies (Figure 10). The spectra analysis revealed a flex point at about pH 3.86 (Figure 10, inset a), which suggests that AgNPs have a negligible influence on the P(PEG350)_3_ pKa value compared to that shown by porphyrin-functionalized gold nanoparticles [36].

To verify the reusability of the hybrid nanosystem as a sensor, the titrated aqueous nanoparticles’ suspension was centrifuged (5000 rpm, 10 min) to remove the nanoparticles. Once separated, they were dried at 80 °C under nitrogen flow and then re-suspended through sonication. During different titration cycles (Figure 10, inset b), negligible performance variations were evidenced, suggesting its potential application as a removable acidity sensor.

**Figure 11:**
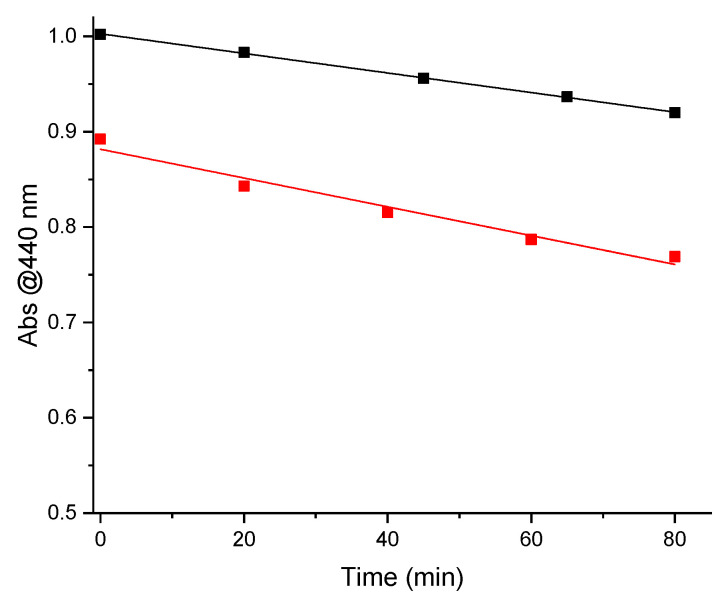
Absorbance variations (λ = 440 nm) of AgNP@P(PEG350)_3_ (black full square) and TPPS (red full square) aqueous solution in function of the irradiation time. The related lines represent the linear fit of the experimental data.

The photosensitizer ability of the AgNP@P(PEG350)_3_ system was investigated determining the quantum yield (Φ) of reactive singlet oxygen species (ROS) generation under laser light irradiation employing a standard method [44]. In particular, due to the bleaching reaction induced by ROS formation, the decrease of the *p*-nitroso-N,N-dimethylaniline (RNO) absorbance value in the presence of AgNP@P(PEG350)_3_ was monitored (Figure 11). The experimental data were compared with those obtained for 5,10,15,20-tetrakis(4-sulfonatophenyl)porphyrin (TPPS) used as a standard reference. Equation (1) revealed a quantum yield (Φ) equal to 0.21 ± 0.05, confirming the photosensitizer properties of the AgNP@P(PEG350)_3_ system.

## 4. Discussion

The covalent coating of silver nanoparticles (AgNPs) represents a fundamental step to tune their features. Such modification allows to influence the surface properties of the materials while not altering the properties of their metallic core. The coupling of metal nanoparticles features with that of PEGylated porphyrin derivatives would allow to obtain advanced nanosystems useful for biomedical applications.

As a first step, the tri-PEGylated porphyrin with a free hydroxyl group (P(PEG350)_3_) was synthesized and isolated.

The AgNPs synthesis was performed in water solution through a silver nitrate reduction mediated by sodium borohydride, producing a stable colloidal suspension of AgNPs having a mean hydrodynamic radius of about 11 nm. Exploiting the thiol affinity for silver, the AgNPs surface was functionalized with 3-chloro-1-propanethiol, leaving the chlorine atoms as the outer nanoparticle shell (AgNP@ClPT). As an effect of this functionalization, the obtained AgNP@ClPT system was not water-soluble, but resulted stable as organic suspension (acetone), revealing a nanoparticle hydrodynamic radius of 25.9 nm.

Then, the AgNP@ClPT system was covalently coated with P(PEG350)_3_ through a nucleophilic substitution involving the AgNP@ClPT chlorine groups and the PEGylated porphyrin hydroxyl groups, producing the AgNP@P(PEG350)_3_ (see Scheme 1). This functionalization combines the spectroscopic features of the PEGylated porphyrin with the typical LSPR signal of the AgNPs. The UV-Vis analysis confirms the overlapping of the P(PEG350)_3_ Soret and Q-bands with the wide LSPR signal of the AgNPs (Figure 4a). Thanks to the PEGylated porphyrin shell, the final hybrid nanosystem was suspendable both in organic and aqueous solvents. Nevertheless, the solvent affects the behavior of the porphyrin moiety. In acetone, UV-Vis spectra show a narrow Soret band, suggesting the absence of aggregation effects. Finally, DLS measurements revealed a mean hydrodynamic radius of about 40 nm. On the contrary, due to inter-particle hydrophobic interactions and solvatochromism of the porphyrin moiety, the AgNP@P(PEG350)_3_ suspended in water showed a broader Soret band. Furthermore, the occurring PEG chains entanglements are reflected in a higher mean hydrodynamic radius than in acetone.

A quantitative result about the organic coating of the AgNPs was obtained through thermogravimetrical analysis, determining an organic loading of about 48% (w).

The TEM characterization of the AgNP@P(PEG350)_3_ confirmed the maintenance of the AgNPs structural properties, revealing the coated nanoparticles’ size values ranging 15–30 nm. Notably, it was also evident as the PEGylated porphyrins, constituting the organic layer, arrange themselves in nanostructured row patterns. As an explanation of the observed structures, the self-organization of these macrocycles guided by π–π stacking interactions between porphyrin cores was considered. The rows’ distance is influenced by the peripheral PEGylated phenylether of the porphyrin, where the polymer chains act as spacers. Considering the length of such organized rows (tens of nanometers), a long-range order of these macrocycles could be hypothesized, as already reported in the literature for this class of molecules [49].

The AgNP@P(PEG350)_3_, combining the peculiar features of P(PEG350)_3_ with that of AgNPs, represents a potential multifunctional nanotool for application in the theranostic field. Particularly, the bactericidal capability and the photothermal properties of AgNPs are coupled with the porphyrin ability to generate reactive singlet oxygen species, together with its sensing ability and strong fluorescence emission. Furthermore, the PEG chains enhance the stealth properties of the system [38]. These features could be potentially exploited in therapies (thanks to their bactericidal capability, photodynamic and photothermal properties) and diagnosis (providing the tracking feature), making this hybrid system a potential theranostic tool.

Since the interaction of the two pyrrolic nitrogen with acids in solution induces strong spectroscopic variations, the hybrid nanosystem revealed itself useful as a sensor to determine the acidity of aqueous media.

To test the H^+^ sensing capability, spectroscopic HCl titrations monitoring both the UV-Vis and the fluorescence signals were performed. Notably, the flex point of the titrations performed on both the (parent) P(PEG750)_4_ and the AgNP@P(PEG350)_3_ were revealed at pH 3.86, confirming the negligible influence of the AgNPs covalent bond onto the porphyrin spectroscopic properties.

This hybrid nanosystem, having a PEG-based outer shell which improves the dispersibility in aqueous solvents, allows the collection and re-suspension of the nanoparticles. Thus, by means of centrifugation and subsequent thermal regeneration, the AgNP@P(PEG350)_3_ showed the fluorescence emission intensity recovery, allowing their use over three titration cycles. Finally, considering the stealth effect provided by the PEG chains that improve the biocompatibility of this nanosystem, their potential application also as a pH sensor for biological environments could be hypothesized.

Finally, to verify the photosensitizer properties of this new system, photobleaching RNO tests were performed to determine the quantum yield of ROS formation (Φ) for AgNP@P(PEG350)_3_ in PBS solution. The calculated value (Φ = 0.21 ± 0.05) is in accordance with a previous calculation for a similar tetra-PEGylated porphyrin system in aqueous solution (Φ = 0.33 ± 0.05) [30]. As a result, the photosensitizers’ properties attributed to the porphyrin moiety are maintained in this hybrid nanosystem, despite the slight decrease of the quantum yield value with respect to the tetra-PEGylated porphyrin. Such an occurrence is due to the proximity of P(PEG350)_3_ moieties bound onto the AgNPs, and has been evidenced in similar hybrid nanosystems [37].

## Data Availability

Data are not available due to further project advancement.

## References

[1] Jain P.K., Huang X., El-Sayed I.H., El-Sayed M.A. (2008). Noble Metals on the Nanoscale: Optical and Photothermal Properties and Some Applications in Imaging, Sensing, Biology, and Medicine. Acc. Chem. Res..

[2] Li H., Xu D. (2014). Silver nanoparticles as labels for applications in bioassays. Trac. Trends Anal. Chem..

[3] Morones J.R., Elechiguerra J.L., Camacho A., Holt K., Kouri J.B., Ramírez J.T., Yacaman M.J. (2005). The bactericidal effect of silver nanoparticles. Nanotechnology.

[4] Sharma V.K., Yngard R.A., Lin Y. (2009). Silver nanoparticles: Green synthesis and their antimicrobial activities. Adv. Colloid Interface Sci..

[5] Nicosia A., Vento F., Pellegrino A.L., Ranc V., Piperno A., Mazzaglia A., Mineo P. (2020). Polymer-Based Graphene Derivatives and Microwave-Assisted Silver Nanoparticles Decoration as a Potential Antibacterial Agent. Nanomaterials.

[6] Ravindran A., Chandran P., Khan S.S. (2013). Biofunctionalized silver nanoparticles: Advances and prospects. Colloids Surf. B Biointerfaces.

[7] Qiang W., Li H., Xu D. (2013). Metal enhanced fluorescent biosensing assays for DNA through the coupling of silver nanoparticles. Anal. Methods.

[8] Li H., Qiang W., Vuki M., Xu D., Chen H.-Y. (2011). Fluorescence Enhancement of Silver Nanoparticle Hybrid Probes and Ultrasensitive Detection of IgE. Anal. Chem..

[9] Lee K.-S., El-Sayed M.A. (2006). Gold and Silver Nanoparticles in Sensing and Imaging: Sensitivity of Plasmon Response to Size, Shape, and Metal Composition. J. Phys. Chem. B.

[10] Lee S., Jun B.-H. (2019). Silver Nanoparticles: Synthesis and Application for Nanomedicine. Int. J. Mol. Sci..

[11] Boca S.C., Potara M., Gabudean A.-M., Juhem A., Baldeck P.L., Astilean S. (2011). Chitosan-coated triangular silver nanoparticles as a novel class of biocompatible, highly effective photothermal transducers for in vitro cancer cell therapy. Cancer Lett..

[12] Khlebtsov B., Panfilova E., Khanadeev V., Bibikova O., Terentyuk G., Ivanov A., Rumyantseva V., Shilov I., Ryabova A., Loshchenov V. (2011). Nanocomposites Containing Silica-Coated Gold–Silver Nanocages and Yb–2,4-Dimethoxyhematoporphyrin: Multifunctional Capability of IR-Luminescence Detection, Photosensitization, and Photothermolysis. ACS Nano.

[13] Li Y., Chang Y., Lian X., Zhou L., Yu Z., Wang H., An F. (2018). Silver Nanoparticles for Enhanced Cancer Theranostics: In Vitro and In Vivo Perspectives. J. Biomed. Nanotechnol..

[14] Samaroo D., Vinodu M., Chen X., Drain C.M. (2007). meso-Tetra(pentafluorophenyl)porphyrin as an Efficient Platform for Combinatorial Synthesis and the Selection of New Photodynamic Therapeutics using a Cancer Cell Line. J. Comb. Chem..

[15] Tessore F., Biroli A.O., Di Carlo G., Pizzotti M. (2018). Porphyrins for Second Order Nonlinear Optics (NLO): An Intriguing History. Inorganics.

[16] Burns J.R., Wood J.W., Stulz E. (2020). A Porphyrin-DNA Chiroptical Molecular Ruler With Base Pair Resolution. Front. Chem..

[17] Mineo P., Micali N., Villari V., Donato M.G., Scamporrino E. (2012). Reading of Protein Surfaces in the Native State at Micromolar Concentrations by a Chirogenetic Porphyrin Probe. Chem. A Eur. J..

[18] Jain R.K., Hamilton A.D. (2002). Designing Protein Denaturants: Synthetic Agents Induce Cytochromec Unfolding at Low Concentrations and Stoichiometries. Angew. Chem. Int. Ed..

[19] Groves K., Wilson A.J., Hamilton A.D. (2004). Catalytic Unfolding and Proteolysis of CytochromecInduced by Synthetic Binding Agents. J. Am. Chem. Soc..

[20] Chung C.Y.-S., Fung S.-K., Tong K.-C., Wan P.-K., Lok C.-N., Huang Y., Chen T., Che C.-M. (2017). A multi-functional PEGylated gold(iii) compound: Potent anti-cancer properties and self-assembly into nanostructures for drug co-delivery. Chem. Sci..

[21] Sibrian-Vazquez M., Jensen T.J., Vicente M.G.H. (2007). Synthesis and cellular studies of PEG-functionalized meso-tetraphenylporphyrins. J. Photochem. Photobiol. B Biol..

[22] Nawalany K., Kozik B., Kepczynski M., Zapotoczny S., Kumorek M., Nowakowska M., Jachimska B. (2008). Properties of Polyethylene Glycol Supported Tetraarylporphyrin in Aqueous Solution and Its Interaction with Liposomal Membranes. J. Phys. Chem. B.

[23] Micali N., Engelkamp H., van Rhee P.G., Christianen P.C.M., Scolaro L.M., Maan J.C. (2012). Selection of supramolecular chirality by application of rotational and magnetic forces. Nat. Chem..

[24] Villari V., Mineo P., Micali N., Angelini N., Vitalini D., Scamporrino E. (2007). Uncharged water-soluble porphyrin tweezers as a supramolecular sensor for α-amino acids. Nanotechnology.

[25] Mineo P., Villari V., Scamporrino E., Micali N. (2014). Supramolecular chirality induced by a weak thermal force. Soft Matter.

[26] Mineo P., Villari V., Scamporrino E., Micali N. (2015). New Evidence about the Spontaneous Symmetry Breaking: Action of an Asymmetric Weak Heat Source. J. Phys. Chem. B.

[27] Waku T., Matsusaki M., Kaneko T., Akashi M. (2007). PEG Brush Peptide Nanospheres with Stealth Properties and Chemical Functionality. Macromolecules.

[28] Farace C., Sánchez-Moreno P., Orecchioni M., Manetti R., Sgarrella F., Asara Y., Peula-García J.M., Marchal J.A., Madeddu R., Delogu L.G. (2016). Immune cell impact of three differently coated lipid nanocapsules: Pluronic, chitosan and polyethylene glycol. Sci. Rep..

[29] Baratta M.G. (2018). Getting to the brain. Nat. Nanotechnol..

[30] Mineo P., Faggio C., Micali N., Scamporrino E., Villari V. (2014). A star polymer based on a polyethylene glycol with a porphyrinic core as a photosensitizing agent for application in photodynamic therapy: Tests in vitro on human erythrocytes. Rsc Adv..

[31] Nicosia A., Vento F., Satriano C., Villari V., Micali N., Cucci L.M., Sanfilippo V., Mineo P.G. (2020). Light-Triggered Polymeric Nanobombs for Targeted Cell Death. Acs Appl. Nano Mater..

[32] Angelini N., Micali N., Mineo P., Scamporrino E., Villari V., Vitalini D. (2005). Uncharged Water-Soluble Co(II)−Porphyrin: A Receptor for Aromatic α-Amino Acids. J. Phys. Chem. B.

[33] Micali N., Mineo P., Vento F., Nicosia A., Villari V. (2019). Supramolecular Structures Formed in Water by Graphene Oxide and Nonionic PEGylated Porphyrin: Interaction Mechanisms and Fluorescence Quenching Effects. J. Phys. Chem. C.

[34] Mineo P.G., Vento F., Abbadessa A., Scamporrino E., Nicosia A. (2019). An optical sensor of acidity in fuels based on a porphyrin derivative. Dye. Pigment..

[35] Gulino A., Mineo P., Bazzano S., Vitalini D., Fragalà I. (2005). Optical pH Meter by Means of a Porphyrin Monolayer Covalently Assembled on a Molecularly Engineered Silica Surface. Chem. Mater..

[36] Mineo P.G., Abbadessa A., Rescifina A., Mazzaglia A., Nicosia A., Scamporrino A.A. (2018). PEGylate porphyrin-gold nanoparticles conjugates as removable pH-sensor nano-probes for acidic environments. Colloids Surf. A Physicochem. Eng. Asp..

[37] Mineo P., Abbadessa A., Mazzaglia A., Gulino A., Villari V., Micali N., Millesi S., Satriano C., Scamporrino E. (2017). Gold nanoparticles functionalized with PEGylate uncharged porphyrins. Dye. Pigment..

[38] Fam S.Y., Chee C.F., Yong C.Y., Ho K.L., Mariatulqabtiah A.R., Tan W.S. (2020). Stealth Coating of Nanoparticles in Drug-Delivery Systems. Nanomaterials.

[39] Mineo P., Scamporrino E., Vitalini D. (2002). Synthesis and Characterization of Uncharged Water-Soluble Star Polymers Containing a Porphyrin Core. Macromol. Rapid Commun..

[40] Vitalini D., Mineo P., Scamporrino E. (1999). Effect of combined changes in delayed extraction time and potential gradient on the mass resolution and ion discrimination in the analysis of polydisperse polymers and polymer blends by delayed extraction matrix-assisted laser desorption/ionization time-of-flight mass spectrometry. Rapid Commun. Mass Spectrom..

[41] Mineo P., Vitalini D., Scamporrino E., Bazzano S., Alicata R. (2005). Effect of delay time and grid voltage changes on the average molecular mass of polydisperse polymers and polymeric blends determined by delayed extraction matrix-assisted laser desorption/ionization time-of-flight mass spectrometry. Rapid Commun. Mass Spectrom..

[42] Scamporrino E., Vitalini D., Mineo P. (1996). Synthesis and MALDI-TOF MS characterization of high molecular weight poly(1,2-dihydroxybenzene phthalates) obtained by uncatalyzed bulk polymerization of O,O’-phthalid-3-ylidenecatechol or 4-methyl-O,O’-phthalid-3-ylidenecatechol. Macromolecules.

[43] Creighton J.A., Blatchford C.G., Albrecht M.G. (1979). Plasma resonance enhancement of Raman scattering by pyridine adsorbed on silver or gold sol particles of size comparable to the excitation wavelength. J. Chem. Soc. Faraday Trans. 2.

[44] Mosinger J., Mička Z. (1997). Quantum yields of singlet oxygen of metal complexes of meso-tetrakis(sulphonatophenyl) porphine. J. Photochem. Photobiol. A Chem..

[45] Villari V., Mineo P., Scamporrino E., Micali N. (2012). Role of the hydrogen-bond in porphyrin J-aggregates. Rsc Adv..

[46] Agnihotri S., Mukherji S., Mukherji S. (2014). Size-controlled silver nanoparticles synthesized over the range 5–100 nm using the same protocol and their antibacterial efficacy. Rsc Adv..

[47] Villari V., Mineo P., Scamporrino E., Micali N. (2012). Spontaneous self-assembly of water-soluble porphyrins having poly(ethylene glycol) as branches: Dependence of aggregate properties from the building block architecture. Chem. Phys..

[48] Gulino A., Mineo P., Scamporrino E., Vitalini D., Fragalà I. (2006). Spectroscopic and Microscopic Characterization and Behavior of an Optical pH Meter Based on a Functional Hybrid Monolayer Molecular System: Porphyrin Molecules Covalently Assembled on a Molecularly Engineered Silica Surface. Chem. Mater..

[49] Gulino A., Mineo P., Fragalà I. (2006). Spectroscopic and Morphological Investigation of an Optical pH Meter Based on a Porphyrin Monolayer Covalently Assembled on a Engineered Silica Surface. J. Phys. Chem. C.

